# Kerala, India's Front Runner in Novel Coronavirus Disease (COVID-19)

**DOI:** 10.3389/fmed.2020.00355

**Published:** 2020-07-02

**Authors:** S. Udhaya Kumar, D. Thirumal Kumar, R. Siva, C. George Priya Doss

**Affiliations:** School of Biosciences and Technology, Vellore Institute of Technology, Vellore, India

**Keywords:** SARS-CoV-2, COVID-19, frontline actions, Kerala (India), epidemic, preventive measures, containment action

## The 21^st^ Century Epidemic Outbreaks in India

**Figure 1:**
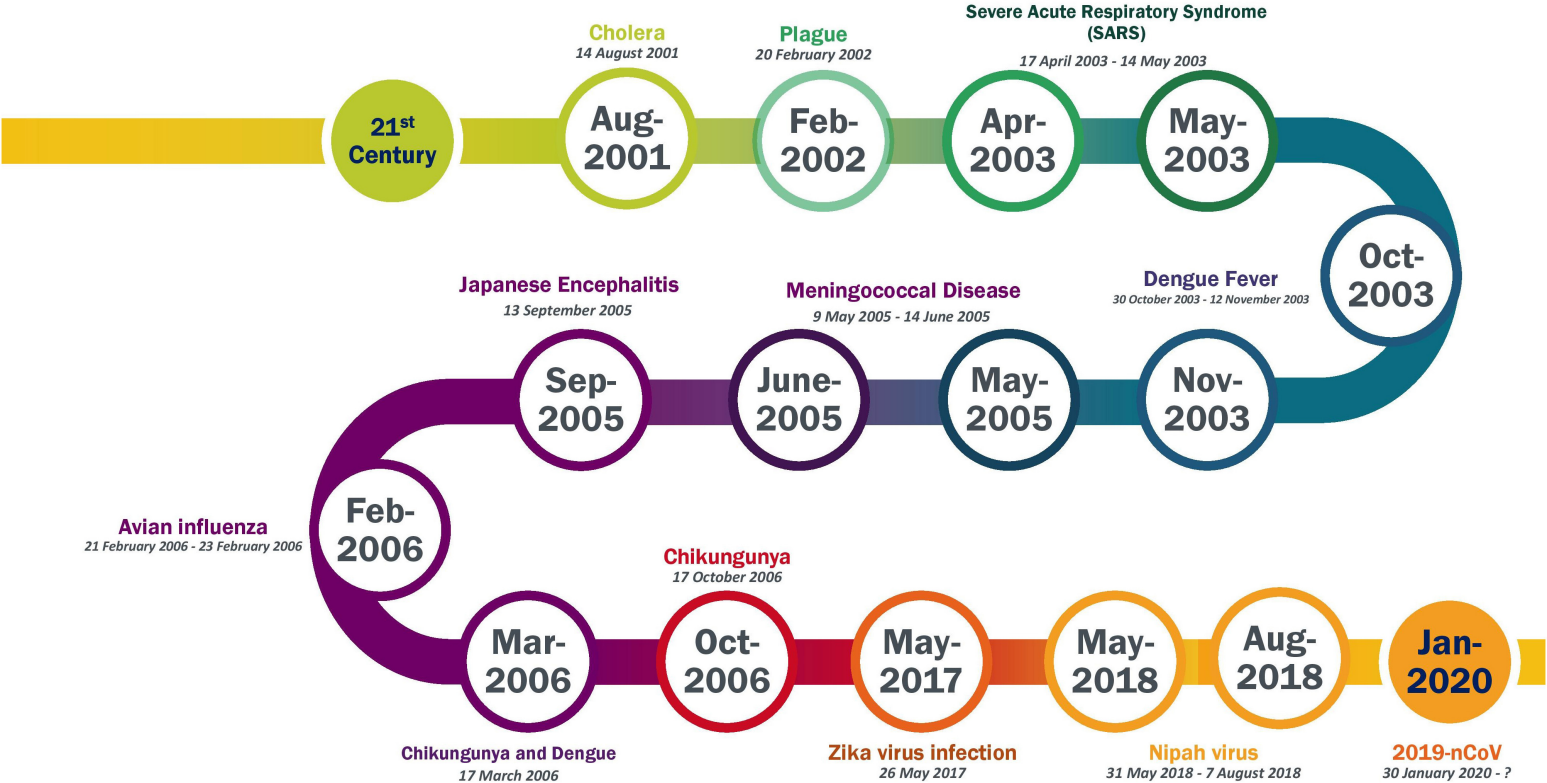
Timeline of epidemic outbreak in India in 21st century. Data Source from WHO website (https://www.who.int/csr/don/archive/country/ind/en/).

Since the beginning of the 21st century, Asian countries have been significantly prone to endemic diseases. This has been particularly true in India due to its increasing population, with the country having faced more than 10 outbreaks, such as Severe Acute Respiratory Syndrome (SARS), Zika virus (ZIKV) disease, and Nipah virus (NiV) disease in the last two decades. A detailed timeline of the outbreaks in India since 21st century is provided in Figure 1. At the beginning of the previous decade (2003–2004), over 8,000 people were infected with Severe Acute Respiratory Syndrome Coronavirus (SARS-CoV), and the death toll had increased to nearly 800 worldwide. At the end of the current decade (2020), the outbreak of the novel and lethal severe acute respiratory syndrome coronavirus 2 (SARS-CoV-2), causing symptoms similar to SARS, has become a pandemic and is threatening humankind. SARS did not spread much in India (1). As per the WHO-Epidemic and Pandemic Alert and Response (EPR) report, only three cases were reported as of July 31, 2003. These cases were reported from the Infectious Diseases Hospital, Kolkata, the Christian Medical College and Hospital (CMCH), Vellore, and Siddhartha Hospital in Pune. No other cases have been reported since then. Notably, reports have stated that 30% of medical doctors and staff from Infectious Diseases Hospital, Kolkata did not work due to fears over infection caused by a lack of sufficient protection (2). All immediate precautionary measures were taken to combat the SARS outbreak in India. Concerning the ZIKV outbreak of 2017, there have been no documented cases of ZIKV infection in India; however, antibodies to ZIKV have been detected in healthy people in India (3). This might have occured as a result of past exposure, although the possibility of cross-reaction with other flaviviruses cannot be denied. The most recent outbreak that India faced was that of NiV disease during mid-2018. As of July 17, 2018, a total of 19 NiV cases, including 17 deaths, had been reported in Kerala State (4, 5). Eighteen of the cases were laboratory-confirmed, and the deceased index case was suspected of having NiV but could not be tested. The outbreak was located in two Kerala districts, Kozhikode and Malappuram. As of July 30, 2018, no new confirmed cases or deaths were reported; NiV transmission from human to human was contained in Kerala.

## Kerala, India Is Vulnerable to Viral and Non-Viral Outbreaks

The state of Kerala, with a total area of 15,005 sq km, is located in the southwestern coastal region of India. According to Census 2011, Kerala has a population of ~36 million, with a literacy rate of 94%, which is the highest in India. The comparatively higher allocation of funds by the Kerala government to primary level education, health care, and the elimination of poverty has resulted in the state being number one in the Human Development Index (HDI) (6). It has resulted in wide recognition of Kerala as the cleanest and healthiest state in the country (7). On the other hand, the state also faced several epidemics. Although the first outbreak of Chikungunya in India was reported in 1963 in Kolkata, after 32 years, the virus reappeared in 2006 in the Alappuzha district of Kerala (8, 9). Various forms of encephalitis, such as Japanese encephalitis (JEV), Acute Encephalitis Syndrome (AES), and West Nile encephalitis (WNV), have been reported in many districts of Kerala. The AES and JEV outbreaks were first reported in 1996 and 1997, with 105 positive cases and 31 deaths, and 121 positive cases and 19 deaths, respectively (10). Reports from the Health Services Directorate (DHS), Kerala, have documented 846, 518, 225, 34, and 191,945 cases of dengue, malaria, leptospirosis (Rat fever), chikungunya, and acute diarrheal disease (ADD), respectively, during a flood that occurred in mid-2018 (Table 1). Rat fever is the leading cause of death in the state, with nearly 1,000 cases being documented annually. Furthermore, an outbreak of NiV occurred recently in Kerala in mid-2018, and 19 cases with a high mortality rate were reported (4). With these previous outbreaks, India's first SARS-CoV-2 infection was identified in Kerala on January 30, 2020, and to date, 497 positive cases have been reported. Among these, 111 cases (22.33%) are still active as of May 1, 2020. With a recovery rate of 77.06% and a mortality rate of 0.6%, the state has 383 recovered cases and three deaths (11). The Kasaragod and Kannur districts bordering the Kerala state have reported the highest number of cases, 179 and 114, respectively.

**Table 1:** List of outbreaks and number of cases in the last two decades in Kerala (2000–2020).

**Outbreaks**	**Cases**	**Death**
Chikungunya	2,720	NA
Japanese encephalitis	59	11
Acute encephalitis syndrome (AES)	286	54
West nile encephalitis	300	4
Dengue	58,919	217
Viral hepatitis	69,284	119
Nipah	19	18
Swine flu (H1N1)	6,553	391
COVID-19	485	4

*The data were obtained from the Central Bureau of Health Intelligence and the National Vector Borne Disease Control Programme (https://cbhidghs.nic.in/index.php and https://nvbdcp.gov.in/index.php)*.

## Effective Containment of Outbreaks in Kerala

After the initial case reports during late January and early February, the Kerala government took numerous steps to strengthen the guidelines, emergency preparedness, diagnostics, and categorization of risk involved in reducing the transmission of the virus in any outbreak. Notably, this state had previously experienced endemics, which helped to deliver a more rigorous action plan during the COVID-19 epidemic as compared to other states in India. Due to its previous epidemic experience, the state immediately declared a health emergency in the first week of February. With no new case reports, the health emergency was withdrawn on February 12, 2020. During this period, all travelers, including student returnees from Wuhan, China, were quarantined, and registrations were initiated to keep a record of travelers entering the state from SARS-CoV-2 affected countries. In mid-February 2020, the number of positive cases gradually increased as many Kerala natives returned from the affected countries. With the recurrence of cases, in mid-March 2020, the state implemented additional precautionary measures, including the immediate shutdown of non-medical educational institutes, surveillance at airports, and the use of sanitizers in public places such as salons, malls, and shopping centers. Further, an immediate fund was released from the State Disaster Response Fund (SDRF) to tackle the outbreak after the identification of COVID-19 as a notified disaster. These precautionary and state lockdowns were more advanced than the national curfew, which was declared 1 week later (March 25, 2020). Despite the severe measures, the entire state was declared to be COVID affected on March 25, 2020. The number of active cases in the state peaked at 266 on April 6 2020. Subsequently, the number of active cases has gradually decreased to date. During the period of lockdown, rigorous testing was performed for symptomatic COVID-19 cases, and contact tracing was carried out for infected people. To date (May 1, 2020), 27,150 samples were tested, out of which 26,225 were found to be negative (data available at https://dashboard.kerala.gov.in/). This was followed by geographical tracking and mapping of confirmed cases, which helped to enable contacts of positive cases to report to the health system and seek advice regarding quarantine. In addition, nearly 82 hotspot regions in Kerala were spotted, and these containment zones are referred to as “LSG Needing Special Attention” (12). The successful containment in this state was due to the effective coordination between the inter-departments at rural and urban levels. In addition, the daily address of the state's Chief Minister helped to instill confidence among the residents. Moreover, the state had deployed many officers from various departments to monitor activities related to containing COVID-19, such as household surveys during the quarantine period. This surveillance at a grassroots level was conducted by local self-government and primary health care workers. As of May 1, 2020, the details of people under surveillance have been provided by the Kerala government. Of these, 21,894, 21,484, and 410 were kept under observation and kept under home isolation, respectively, and 410 symptomatic persons were hospitalized (13). If domestic flights resumed, it is mandatory for travelers to use the Covid19 Jagratha portal (https://covid19jagratha.kerala.nic.in/) to register their information and agree to the quarantine norms (14). After medical examination for any COVID-19 symptoms, asymptomatic travelers were requested to follow home quarantine while those with symptoms are referred to either a hospital or a COVID care center.

The Kerala state governor implemented the Kerala Epidemic Diseases Ordinance on March 26, 2020. This regulation authorizes the government to take any required steps and legislations to combat the risk of COVID-19 disease. In the absence of medicine to treat COVID-19, existing allopathic medicines were used as repurposed drugs. In addition, AYUSH departments were included in the outbreak preparedness and containment activities. Moreover, Kerala efficiently used a traditional medicine “Triphala” against the Hepatitis A outbreak. Likewise, Ayurveda supplements were provided to develop immunity against COVID-19 (15, 16). At this point in time, the states of Punjab and Karnataka had an approximate number of COVID-19 cases of 186 and 277, respectively (11). However, the number of cases gradually increased later in these states compared to Kerala, highlighting the efficient control of virus transmission in the state of Kerala (11).

With the highest literacy rate, it became easy for the state government to generate awareness for COVID-19. Furthermore, the state restricted the movement of migrant workers to other places by constructing shelters and provided food for thousands due to immediate national full lockdown. To date, 1,034 local self-government institutions (LSGIs), along with 853 community kitchens (CK), are actively working to ensure Kerala is hunger-free during the lockdown. The CK has served food to nearly 86,51,627 individuals in Kerala (12). Psychosocial Support (PSS) calls and counseling services are being provided to people with psychiatric issues, senior citizens, guest workers, and children with special care needs. So far, 9,06,365 PSS and counseling calls have been provided by 1,107 PSS personnel (12). The state also took advantage of technology and launched a mobile app, “GoK Direct,” which releases important information on COVID-19. Moreover, the state has established “walk-in facility” centers for people to safely test for SARS-CoV-2 infection (https://dashboard.kerala.gov.in/). In line with this, the state also established a “CoronaSafe Network,” which mediates two essential components that includes “Corona Care Centre” and “Corona literary Mission” to ensure COVID-19 awareness amongst its people. The government of Kerala has also started telemedicine consultation services for Keralites across many countries, which can be used to provide consultation to SARS-CoV-2 infected patients following discharge. This immediate taskforce management and preventive measures taken by the Kerala government aided in maintaining a low rate of mortality in Kerala.

## Author Contributions

SU, DT, and CG were involved in the design of the study and drafting of the manuscript. CG and RS supervised the entire study. The manuscript was reviewed and approved by all the authors.

## Conflict of Interest

The authors declare that the research was conducted in the absence of any commercial or financial relationships that could be construed as a potential conflict of interest.
